# Positive Youth Development Attributes and Parenting as Protective Factors Against Adolescent Social Networking Addiction in Hong Kong

**DOI:** 10.3389/fped.2021.649232

**Published:** 2021-03-18

**Authors:** Lu Yu, Daniel Tan Lei Shek

**Affiliations:** Department of Applied Social Sciences, The Hong Kong Polytechnic University, Kowloon, Hong Kong

**Keywords:** parenting, positive youth development, public health, middle school, networks, problem/risky/antisocial behaviors

## Abstract

This study examined the predictive effects of 15 positive youth development (PYD) attributes and parenting behavior on adolescent social networking addiction (SNA) in a representative sample of Hong Kong students. In total, 1,896 Hong Kong Secondary 1 students from 20 randomly selected schools (age = 13.19 ± 0.52 years) completed the Bergen Social Media Addiction Scale (BSMAS), the Chinese Positive Youth Development Scale, and the Chinese Parenting Behavior Scale. Of the participants, 11.4% could be classified as being addicted to Social Networking Sites (SNSs). Regression analyses showed that students' emotional competence (β = −0.09; *p* < 0.01), behavioral competence (β = −0.12; *p* < 0.001), beliefs in the future (β = −0.10; *p* < 0.01), and spirituality (β = −0.08; *p* < 0.01) were negatively associated with SNA, while social competence (β = 0.07; *p* < 0.05) and positive identity (β = 0.13; *p* < 0.001) were positively related to SNA. Paternal and maternal responsiveness showed indirect effects on students' SNA through the full mediation of PYD attributes. Specific PYD attributes and positive parenting behavior may serve as important protective factors against the development of SNA among Hong Kong adolescents.

## Introduction

Online social networking sites (SNSs) such as Facebook, Instagram, and Twitter are becoming increasingly popular. In 2019, there were 3.72 billion active SNS users worldwide (1). In Hong Kong, social media participation in the general population increased from 68% in 2014 to 83% in 2018, and by 2019 over 90% of youngsters aged 10–24 were actively using SNSs (2). Young people aged 15–24 are reportedly the heaviest users, spending 17.7 h on SNSs per week (2.5 h per day) on average. SNSs have become an indispensable part of young people's daily lives in Hong Kong.

The use of SNSs may be beneficial as it allows one to communicate with other people globally in a convenient manner. Researchers have reported evidence of a broad range of benefits to young people associated with the use of SNSs, such as media literacy, creativity, positive education outcomes, civic participation, and well-being (3). There are also findings showing that support and social bond available from online social networking can reduce depressive symptoms, especially during COVID-19 (4, 5).

However, intensive involvement in online social networking activities increases the risk of developing problematic SNS use or social networking addiction (SNA) (6). Ample evidence exists to suggest that SNA may lead to a variety of negative psychosocial and physical outcomes (5, 7). Researchers also found that adolescents are particularly susceptible to SNA (8) due to their unique developmental characteristics. Therefore, understanding how SNA develops in adolescents and the factors associated with it are critically important to address this problem effectively.

While many studies have evaluated SNA prevalence in adolescents worldwide and the associated correlates (9), there is a lack of a reliable SNA prevalence rate among Hong Kong adolescents. Most SNA research targets university students and uses convenience samples [e.g., (10)]. Among the limited studies, Facebook addiction has been the major focus (11), with no systematic study of the excessive use of other popular social networking sites and applications such as Twitter, WeChat, or Instagram among young people in Hong Kong. Furthermore, existing theories about the formation of SNA have focused primarily on risk factors that increase the possibility of developing SNA from an etiological perspective. Few attempts have been made to investigate the role of different protective factors at the individual and environmental level that may prevent the occurrence of SNA.

In the present study, our first aim is to examine the prevalence of SNA in Hong Kong adolescents with a representative sample of secondary students using a psychometrically sound instrument widely used in other countries and validated in our target population. Second, we seek to investigate the protective effects of positive personal attributes and parenting behavior on adolescent SNA based on a positive psychology theoretical framework.

Social Networking Addiction has been defined as “being overly concerned about SNSs, to be driven by a strong motivation to log on to or use SNSs, and to devote so much time and effort to SNSs that it impairs other social activities, studies/job, interpersonal relationships, and/or psychological health and well-being” (6). Kuss and Griffiths (8) further described SNA as uncontrollable online social networking behaviors with six major characteristics of behavioral addiction: “salience,” “mood modification,” “tolerance,” “withdrawal,” “relapse,” and “problems arise as a consequence of one's compulsive SNSs use.” Although the DSM-5 does not include SNA as a formal diagnosis, growing evidence shows that SNA is associated with many negative outcomes, including academic dysfunction (12), insomnia (13), deteriorating interpersonal relationships (14), low self-esteem, and life satisfaction (15), as well as mental health problems such as anxiety and depression (16).

It is worth noting that adolescents may be particularly vulnerable to SNA due to their developmental characteristics (17). Researchers have suggested that two major tasks during adolescence are “to develop an identity and pursue autonomy” and “to find comfortable affiliations and gain acceptance from peers” [(18), p. 48]; SNS use offers adolescents new and convenient opportunities to explore both (19). Those who are uncomfortable interacting with peers face-to-face are more likely to engage in excessive online social networking to meet their social needs, as the internet provides a safer and more familiar place for interactions (20–22). It is also understood that adolescents have relatively low self-control over their behavior. Given these characteristics and the extreme popularity of SNSs among young people, researchers have warned that SNA in youth has become an emergent public health issue (8). In fact, the reported high prevalence rates of youth internet addiction [17.9–26.4%; (23)] and mobile phone addiction [27.4%; (24)] in Hong Kong suggest that the percentage of adolescents with SNA may also be high. However, a systematic investigation into the prevalence of adolescent SNA in Hong Kong has rarely been conducted based on representative samples.

Several models have been developed to explain the formation of SNA, mainly based on existing theories of behavioral and substance-related addictions [e.g., (25)]. Generally, SNA has been conceptualized as a specific form of internet addiction. A recent review of major theoretical models in the field (20, 26–28) suggested that SNA is fostered by an integration of risk factors at biological (e.g., impulsivity), demographic (e.g., gender, age), psychological (e.g., narcissism, lack of social skills), and socio-cultural (e.g., family dynamics) levels. One problem with the current SNA models is that SNA is viewed mainly from an etiological perspective, so risk factors that increase the possibility of developing SNA are the predominant focus of many related empirical studies, whereas little is known about what biopsychosocial factors may protect individuals from developing SNA [e.g., (26)]. The promotion of protective factors is as important as reducing risk factors in prevention science, as protective factors mitigate risk and promote healthy development and well-being, and are likely to contribute to both short-term and long-term positive outcomes (29). Simply eliminating risk factors does not achieve this (30).

However, current SNA research has largely overlooked protective factors, leading to an incomplete understanding of the overall problem, and hindering effective work in the prevention of SNA on the basis of both risk and protective factors. Thus, the present study aims to examine protective factors in the development of SNA.

With the emergence of positive psychology, there has been a paradigm shift in prevention science away from focusing on single problems among a specific group of young people, to promoting strength building to enable adolescents to overcome adversity and deal with different problems (31, 32), known as the PYD approach (33). This framework emphasizes that young people are resources to be developed, and that problematic behavior is “only one instance of a theoretically larger array of outcomes that include the possibility of positive development” [(34), p. 10]. Based on PYD, scholars have identified a set of competences, experiences, and relationships as building blocks of healthy development, referred to as developmental assets. In the past two decades, some empirical studies have supported the notion that different developmental assets serve as significant protective factors in risk behavior (32). PYD offers a unique and supplementary lens to understand SNA and its associated protective factors.

According to Benson (31), developmental assets can be further sub-categorized into internal assets possessed by individuals (e.g., resilience, interpersonal competence) and external assets of environmental protective factors (e.g., family support, positive peer influences) that nurture positive development.

Catalano et al. (32) summarized 15 PYD attributes (bonding, resilience, cognitive competence, social competence, emotional competence, behavioral competence, moral competence, self-determination, self-efficacy, clear and positive identity, spirituality, belief in the future, pro-social norms, pro-social involvement, and recognition for positive behavior) based on a systematic review of effective youth prevention programmes. According to Catalano et al. (32), the PYD attributes help in understanding “the developmental precursors of both positive and negative youth development” (p. 100). Nonetheless, the relationships between these PYD attributes and SNA have not been systematically examined. In light of the synthesized theory of change (35), developmental assets reduce problematic behavior through either compensating for the risks taken by adolescents, or buffering against risks in their environment. Buffering refers to the effect of positive assets giving environmental risk factors a less negative outcome on behavior, while compensation focuses on the effect of positive assets that allow for less harmful consequences of risk behavior already engaged in. It has also been posited that different assets may reduce risk in a rather molecular way (i.e., a specific asset offers protection against a specific risk), or through “pile-up”—the accumulation of various assets would likely lead to the reduction of problems.

Sporadic evidence in the literature suggests that PYD attributes may have both buffering and compensating effects in SNA prevention. For example, social learning theory (36) indicates that adolescents' SNS behavior can be reinforced by peer pressure and modeling, while adolescents with positive identities have been found to be less affected by peer influence (32). As such, positive identity may buffer the negative effects of peer influence on SNA. In terms of compensating effects, it is likely that teenagers who have positive relationships with adults (bonding) may still use SNSs frequently, but with fewer negative consequences, as the existing positive relationship helps to solve potential conflict (6). There is also evidence showing that socially skilled adolescents use SNSs particularly to benefit from SNS use, by trying out new identities, learning new social skills, and establishing affiliations (19). Moreover, some risk factors identified in the syndrome-based model also provide indirect evidence for the protective effects of PYD attributes on SNA, in both molecular and accumulative ways. For instance, the finding that adolescents with SNA often show high neuroticism and low self-esteem levels indicates that building a positive identity and developing emotional competence in adolescents would help reduce SNA (37). However, there is a lack of direct evidence on the influence and relative importance of different PYD attributes on SNA. To fill this gap, this study examined the predictive effects of Catalano et al.'s (32) 15 PYD attributes on adolescent SNA. Based on previous literature (8, 23, 38), it was hypothesized that the PYD attributes would predict reduced SNA.

Apart from the PYD attributes, positive parenting behavior has long been considered as an important external developmental asset (31) with a pivotal role in adolescents' optimal development. The effects of parenting behavior on young people's addictive behavior have been widely reported, including in addiction studies. For instance, low levels of parental monitoring has been associated with a higher risk of gambling addiction (39), while enhanced parent-adolescent attachment was related to a lower motivation among adolescents to gamble (40).

However, few studies have been published on the role of parenting behavior with reference to adolescent SNA. This may be due to the fact that SNA is a relatively new phenomenon and most of the existing studies in the field are based on university students (6), for whom the effects of parental factors were considered as less important than other factors, such as peers and personal traits. Yet for adolescents, particularly secondary school students, who remain living with family members, parental influence on their behavior is still prominent. Drawing on previous evidence about the protective effects of positive parenting behavior on other types of youth addiction, it can be assumed that positive parenting may also result in reduced SNA.

In the present study, we adopted Baumrind's (41) two-dimensional model of parenting styles (demandingness and responsiveness) to investigate parenting behavior; a model that has been validated in Hong Kong (42). Parental responsiveness refers to “the extent to which parents intentionally foster individuality, self-regulation, and self-assertion by being attuned, supportive, and acquiescent to children's special needs and demands” [(41), p. 410]. Parental demandingness has been defined as “the claims parents make on children to become integrated into the family whole by their maturity demands, supervision, disciplinary efforts and willingness to confront the child who disobeys” [(41), p. 411]. Both theory and empirical studies have suggested that parental responsiveness and demandingness are protective factors in youth development (42, 43). We therefore hypothesized that parental responsiveness and demandingness would directly predict adolescent SNA negatively.

Moreover, positive parenting behavior contributes to the development of different PYD attributes. There is evidence that adolescents raised by parents with high parental responsiveness and demandingness show a variety of positive outcomes in diverse domains, including increased competence, a more positive identity, strong attachments with parents and peers, higher social competence, more developed behavioral and emotional autonomy, greater success in school, increased curiosity, creativity, and reasoning skills, as well as enhanced empathy and moral judgment [e.g., (44)]. As such, we hypothesized that positive parenting behavior would have indirect effects on adolescent SNA through PYD attributes.

The current study had two major aims. First, we aimed to examine the prevalence of social networking addiction among Hong Kong adolescents and to provide a descriptive profile of youth social networking behavior based on a representative sample of Hong Kong junior secondary school students. Second, we intended to investigate the protective role of positive youth development (PYD) attributes and positive parenting behavior in preventing social networking addiction. Specifically, it was hypothesized that PYD attributes would have direct and negative effects on adolescent social networking addiction, and that positive parenting behavior (paternal responsiveness and demandingness, maternal responsiveness, and demandingness) would have both direct and indirect mitigating effects on adolescent social networking addiction through PYD attributes.

## Methods

### Participants

The target population was Hong Kong local junior secondary school students. According to Educational Bureau of Hong Kong SAR (45), there are a total of 504 secondary schools in Hong Kong, with 471 local schools and 33 international schools. Most students (94.5%) were enrolled in local schools, with a male to female ratio of 1.06:1. To obtain a representative sample, two-stage cluster sampling was adopted, with 20 schools randomly selected from all Hong Kong local secondary schools. In these schools, all students in Secondary 1 (i.e., Grade 7) were invited to participate in the study. A total of 1,896 students aged between 10 and 16 years (mean = 13.19 ± 0.52 years) participated in the survey (95.5% response rate), of whom 985 were male (52%) and 911 female (48%). Parental educational level was used to indicate the participants' socio-economic status. Most students reported that their parents had received secondary education (56% both fathers and mothers), followed by those whose parents had received university education or higher (26% mothers, 29.2% fathers), comparable to the general educational attainment of the Hong Kong population (46).

### Procedure

After obtaining the requisite ethical approval and informed consent from the participating schools, an invitation letter consisting of an information sheet and a consent form were sent to parents of the students via the participating schools' internal e-notice system. Parents who agreed with their children's participation in the study provided their e-signature on the consent form and submitted it to the system. Only students with parental consent were invited to participate in the survey. The purpose of the study and confidentiality of the data were explained to students, who were also assured that their participation was voluntary and anonymous. After obtaining written consent from the students, the paper-and-pencil questionnaire was administered by trained researchers in classrooms, taking about 30 min to complete. The research staff collected the students' responses immediately after the survey. Class teachers and other school staff were absent during the entire data collection process. Data collected from the questionnaires were handled by members of the Research Team only. Data files without personal identifiers were securely stored and only members of the Research Team were able to gain access to. We only reported the aggregated results rather than the information of the single participant.

### Measures

#### Bergen Social Media Addiction Scale (BSMAS)

The participants' SNA was measured using the validated Chinese 6-item BSMAS (47, 48). The BSMAS measures six core SNA symptoms: salience, tolerance, mood modification, relapse, withdrawal, and conflict. For each item, participants rated the frequency of the stated problematic behavior on a 5-point scale (1, very rarely; 5, very often) based on their experiences in the past year. According to Bányai et al. (9), a score of 19 or above indicates that an individual is at risk of SNA. Cronbach's alpha coefficient for the BSMAS was 0.88. As well as problematic social networking behavior measured by BSMAS, we also estimated students' SNS usage with two additional items: daily time spent on SNSs in the last month (0, no use; 1, less than an hour; 2, 1 h; 3, 2–3 h; 4, 4–5 h; 5, more than 6 h) and self-perceived SNS addiction (1, not addicted at all; 2, not really addicted; 3, slightly addicted; 4, fairly addicted; 5, very addicted). These two items can provide information on adolescents' actual use of SNSs in daily life and their awareness of social networking addiction. Comparing the at-risk group identified by BSMAS with the low risk group in these two item scores also provides evidence for the validity of the cut-off criterion of the scale.

#### Chinese Positive Youth Development Scale (CPYDS)

The CPYDS is a comprehensive questionnaire measuring multiple areas of Chinese adolescents' PYD (49). We adopted this measure to assess 15 PYD attributes: bonding (3 items), resilience (3 items), emotional competence (3 items), social competence (3 items), cognitive competence (3 items), behavioral competence (3 items), moral competence (3 items), self-determination (3 items), beliefs in the future (3 items), self-efficacy (2 items), clear and positive identity (3 items), pro-social norms (3 items), spirituality (3 items), pro-social involvement (3 items), and recognition for positive behavior (3 items). Items were rated on a 6-point Likert scale (1, strongly disagree; 6, strongly agree).

Previous studies based on Confirmatory Factor Analysis (CFA) have supported the factorial structure of the CPYDS (50). In the present study, the 15-factor model was further examined and the results of CFA provided an acceptable fit to the data, *X*^2^ = 4344.05, *df* = 801, *p* < 0.001; CFI = 0.93, TLI = 0.92, RMSEA = 0.048 (0.047–0.050), and SRMR = 0.04. All factor loadings were above 0.60. Cronbach's alpha coefficients for the 15 subscales ranged from 0.68 (self-efficacy) to 0.89 (spirituality), indicating acceptable to good internal consistency.

#### Chinese Parenting Behavioral Scale (CPBS)

The validated 20-item CPBS (51) developed by Shek (52) was adopted to measure general parenting behavior regarding parental responsiveness (13 items) and parental demandingness (7 items). Items were rated on a 4-point Likert scale (1, strongly disagree; 4, strongly agree). Paternal and maternal behavior were rated separately. Higher scores indicated higher levels of positive parenting behavior. The CPBS has shown good reliability and validity in measuring parenting behavior among different Chinese populations (53). In the present study, we tested the two-factor structure (responsiveness and demandingness) of the paternal behavior scale and the maternal behavior scale separately. CFA results showed good model fitting for both scales, *X*^2^ = 980.79, *df* = 158, *p* < 0.001, CFI = 0.95, TLI = 0.94, RMSEA = 0.05 (0.05–0.06), and SRMR = 0.05 for paternal behavior; *X*^2^ = 932.93, *df* = 155, *p* < 0.001, CFI = 0.95, TLI = 0.94, RMSEA = 0.051 (0.048–0.055), and SRMR = 0.06 for maternal behavior. Cronbach's alpha coefficients were 0.87, 0.86, 0.76, and 0.74 for paternal responsiveness, paternal demandingness, maternal responsiveness, and maternal demandingness, respectively.

### Data Analyses

Descriptive statistics of respondents' SNA behavior were first calculated to provide an overall picture of SNA prevalence and daily time spent on SNSs in Hong Kong adolescents. Pearson's product-moment correlation coefficients were then computed among all the variables under study. To examine the hypothesized relationships among PYD attributes, positive parenting behavior, and adolescent SNA, a series of multiple regression analyses were performed.

The first regression model was to examine the direct effects of parenting behavior on SNA, in which SNA served as the dependent variable; age and gender were entered in the first block, and the four parenting behaviors (i.e., paternal responsiveness, paternal demandingness, maternal responsiveness, and maternal responsiveness) were entered in the second block. The second regression model examined the direct effects of PYD attributes and parenting behavior on SNA simultaneously. The procedure was similar to the first model except that in the second block both parenting behavior and the 15 PYD attributes were entered as predictors.

Another 15 regression models were tested to examine the predictive effects of parenting behavior on the 15 PYD attributes. In each model, one PYD attribute (e.g., bonding) served as the dependent variable, and four parenting behaviors (i.e., paternal responsiveness, paternal demandingness, maternal responsiveness, and maternal responsiveness) were entered simultaneously as the predictors.

## Results

### Students' Problematic Social Networking Behavior

Table 1 shows the numbers and percentages of students reporting problematic social networking behavior at different frequencies as measured by the BSMAS. A significant proportion of students responded at least “sometimes” to some problematic social networking behavior in the past year. Over half (53.6%) reported that they “spent a lot of time thinking about social networking websites or applications or planned use of them” (i.e., salience) either sometimes or more frequently. Around one-third at least “sometimes” “felt an urge to use networking websites or applications more and more” (35.7%; tolerance) or “used networking websites or applications so much that it had a negative impact on their job or studies” (34.5%; conflict). Also, over 30% “used networking websites or applications in order to forget about personal problems”—indicating those adolescents using SNSs as an escape from problems. Students' BSMAS scores ranged between 6 and 30, with a mean score of 12.81 (*SD* = 5.00). Adopting Bányai et al.'s (9) criterion, 213 students scored 19 or above, suggesting an SNA prevalence rate of 11.4%.

**Table 1:** Numbers and percentages of participants reporting SNA behavior at different frequencies measured by the BSMAS.

**BSMAS items**	**(1) Very rarely**	**(2) Rarely**	**(3) Sometimes**	**(4) Often**	**(5) Very often**	**Combined (4 + 5)**
Spent a lot of time thinking about social networking websites or applications, or planned use of them.	352 (18.6%)	526 (27.8%)	724 (38.2%)	221 (11.7%)	70 (3.7%)	291 (15.4%)
Felt an urge to use networking websites or applications more and more.	555 (29.4%)	660 (35.0%)	500 (26.5%)	130 (6.9%)	43 (2.3%)	173 (9.2%)
Used networking websites or applications to forget about personal problems.	709 (37.6%)	584 (30.9%)	384 (20.3%)	152 (8.1%)	58 (3.1%)	210 (11.2%)
Tried to cut down on the use of networking websites or applications without success.	777 (41.1%)	532 (28.1%)	411 (21.7%)	124 (6.6%)	46 (2.4%)	170 (9.0%)
Became restless or troubled if prohibited from using networking websites or applications.	853 (45.2%)	569 (30.1%)	327 (17.3%)	90 (4.8%)	50 (2.6%)	140 (7.4%)
Used networking websites or applications so much that it had a negative impact on job or studies.	689 (36.5%)	547 (29.0%)	459 (24.3%)	114 (6.0%)	79 (4.2%)	652 (10.2%)
In the past month, how much time per day have you spent on social networking sites or applications?	(1) < 1 h	(2) 1 h	(3) 2–3 h	(4) 4–5 h	(5) More than 6 h	Combined (4 + 5)
	555 (29.2%)	426 (22.5%)	564 (29.7%)	238 (12.6%)	113 (6.0%)	351 (18.6%)
To what extent do you perceive yourself as being addicted to social networking sites or applications?	(1) Not at all	(2) Not really addicted	(3) Slightly addicted	(4) Fairly addicted	(5) Highly addicted	Combined (4 + 5)
	297 (15.7%)	545 (28.8%)	675 (35.7%)	298 (15.7%)	78 (4.1%)	376 (19.8%)

Regarding reported daily SNS time, 29.6% reported that they had spent 2–3 h daily on SNSs in the past month, while 12.6 and 6.0% respectively spent 4–5 h and 6 or more hours daily on SNSs. Over one-third (35.7%) perceived themselves as slightly addicted, 15.7% as fairly addicted, and 4.1% as highly addicted to SNSs. Independent sample *t*-tests showed that students of the SNA group identified by BSMAS scored significantly higher on daily time spent on SNSs (*t* = −14.23, *p* < 0.001) and self-perceived addiction to SNSs (*t* = −18.29; *p* < 0.001) than did the non-SNA group: M = 4.48 ± 1.23 vs. M = 3.26 ± 1.17 for daily time, and M = 3.78 ± 0.94 vs. M = 2.49 ± 0.98 for perceived SNA, supporting the validity of the BSMAS and of Bányai et al.'s (9) cut-off criterion.

### Descriptive Statistics

Table 2 shows the descriptive statistics of Pearson's correlation coefficients among all key variables. Parenting behavior was positively associated with all PYD attributes and negatively related to SNA (*ps* < 0.01). PYD attributes were negatively related to SNA (*ps* < 0.01). SNA was also positively related to age and negatively related to gender. The significant correlations were consistent with our hypotheses; however, it should be noted that no causal inferences can be made.

**Table 2:** Descriptive statistics and simple correlation coefficients among variables.

	**Mean**	***SD***	**1**	**2**	**3**	**4**	**5**	**6**	**7**	**8**	**9**	**10**	**11**	**12**	**13**	**14**	**15**	**16**	**17**	**18**	**19**	**20**	**21**
1. Age	13.19	0.52	–																				
2. Gender	0.52	0.50	0.08^**^	–																			
3. Paternal responsiveness	0.95	0.42	−0.09^**^	0.01	–																		
4. Paternal demandingness	1.23	0.62	−0.12^**^	−0.14^**^	0.60^**^	–																	
5. Maternal responsiveness	1.49	0.57	−0.12^**^	0.00	0.51^**^	0.29^**^	–																
6. Maternal demandingness	1.29	0.39	−0.12^**^	−0.20^**^	0.27^**^	0.57^**^	0.54^**^	–															
7. Bonding	4.64	0.91	0.02	−0.02	0.40^**^	0.24^**^	0.37^**^	0.21^**^	–														
8. Resilience	4.57	0.94	0.05^*^	0.03	0.25^**^	0.14^**^	0.28^**^	0.14^**^	0.58^**^	–													
9. Social competence	4.75	0.91	0.00	−0.04	0.24^**^	0.14^**^	0.22^**^	0.12^**^	0.54^**^	0.59^**^	–												
10. Recognition for positive behavior	4.35	0.98	0.04	−0.05^*^	0.31^**^	0.19^**^	0.27^**^	0.14^**^	0.69^**^	0.53^**^	0.56^**^	–											
11. Emotional competence	4.34	0.97	−0.01	−0.50^*^	0.24^**^	0.14^**^	0.23^**^	0.13^**^	0.50^**^	0.59^**^	0.62^**^	0.53^**^	–										
12. Cognitive competence	4.44	0.91	0.03	0.00	0.25^**^	0.11^**^	0.23^**^	0.10^**^	0.45^**^	0.60^**^	0.56^**^	0.49^**^	0.70^**^	–									
13. Behavioral competence	4.58	0.86	0.01	−0.03	0.23^**^	0.14^**^	0.21^**^	0.12^**^	0.49^**^	0.57^**^	0.59^**^	0.52^**^	0.66^**^	0.68^**^	–								
14. Moral competence	4.45	0.90	0.05^*^	−0.11^**^	0.23^**^	0.16^**^	0.26^**^	0.17^**^	0.48^**^	0.56^**^	0.49^**^	0.49^**^	0.58^**^	0.63^**^	0.62^**^	–							
15. Self-determination	4.49	0.95	0.02	0.04	0.27^**^	0.16^**^	0.24^**^	0.13^**^	0.46^**^	0.58^**^	0.56^**^	0.45^**^	0.57^**^	0.63^**^	0.62^**^	0.59^**^	–						
16. Self-efficacy	4.45	0.98	0.04	0.02	0.25^**^	0.14^**^	0.24^**^	0.13^**^	0.46^**^	0.57^**^	0.50^**^	0.46^**^	0.49^**^	0.54^**^	0.54^**^	0.53^**^	0.67^**^	–					
17. Clear and positive identity	4.10	1.12	0.06^*^	0.10^**^	0.32^**^	0.16^**^	0.30^**^	0.12^**^	0.52^**^	0.61^**^	0.60^**^	0.54^**^	0.59^**^	0.57^**^	0.56^**^	0.52^**^	0.68^**^	0.66^**^	–				
18. Beliefs in the future	4.36	1.12	0.03	0.11^**^	0.32^**^	0.19^**^	0.32^**^	0.16^**^	0.48^**^	0.63^**^	0.51^**^	0.46^**^	0.50^**^	0.57^**^	0.51^**^	0.51^**^	0.64^**^	0.60^**^	0.75^**^	–			
19. Prosocial involvement	4.45	0.99	0.01	−0.06^**^	0.33^**^	0.21^**^	0.32^**^	0.19^**^	0.68^**^	0.57^**^	0.56^**^	0.68^**^	0.56^**^	0.54^**^	0.52^**^	0.51^**^	0.50^**^	0.52^**^	0.59^**^	0.55^**^	–		
20. Prosocial norms	4.60	0.91	−0.03	−0.12^**^	0.25^**^	0.20^**^	0.29^**^	0.23^**^	0.55^**^	0.49^**^	0.44^**^	0.53^**^	0.50^**^	0.49^**^	0.50^**^	0.59^**^	0.45^**^	0.43^**^	0.42^**^	0.46^**^	0.62^**^	–	
21. Spirituality	4.92	1.46	0.01	0.03	0.37^**^	0.23^**^	0.35^**^	0.19^**^	0.48^**^	0.58^**^	0.44^**^	0.44^**^	0.45^**^	0.41^**^	0.39^**^	0.37^**^	0.42^**^	0.43^**^	0.56^**^	0.55^**^	0.51^**^	0.40^**^	
22. Social networking addiction	12.81	5.00	0.09^**^	−0.06^**^	−0.17^**^	−0.13^**^	−0.11^**^	−0.08^**^	−0.16^**^	−0.17^**^	−0.12^**^	−0.13^**^	−0.19^**^	−0.17^**^	−0.19^**^	−0.15^**^	−0.16^**^	−0.13^**^	−0.14^**^	−0.19^**^	−0.16^**^	−0.16^**^	−0.21^**^

* p < 0.05 and

** *p < 0.01*.

*Gender: 0, female; 1, male*.

### Predictive Effects of PYD Attributes and Parenting Behavior on SNA

Table 3 summarizes the results of the multiple regression analysis examining the direct effects of parenting behavior on SNA after controlling for the effects of age and gender (Model 1). First, participants' age was positively related to their BSMAS score (β = 0.08; *p* < 0.001), while being male was negatively associated with SNA (β = −0.06; *p* < 0.01). Older adolescents and female students tended to display more SNA behavior. Second, paternal responsiveness was negatively associated with SNA (β = −0.13; *p* < 0.001). The higher the father's responsiveness, the less social networking behavior the adolescent displayed.

**Table 3:** Predictive effects of parenting behavior on SNA before and after adding PYD attributes and factors.

**Model 1**	**B**	**SE**	**β**	***ΔR*^**2**^**	**Model 2**	**B**	**SE**	**β**	***ΔR*^**2**^**
**Block1**				0.01^***^	**Block1**				0.01^**^
Age	0.80	0.24	0.08^**^		Age	0.78	0.26	0.08^**^	
Gender	−0.60	0.25	−0.06^*^		Gender	−0.43	0.27	−0.05	
**Block 2**				0.04^***^	**Block 2**				0.07^***^
Paternal responsiveness	−1.55	0.44	−0.13^***^		Paternal responsiveness	−0.69	0.50	−0.06	
Paternal demandingness	−0.32	0.31	−0.04		Paternal demandingness	−0.30	0.33	−0.04	
Maternal responsiveness	−0.28	0.46	−0.02		Maternal responsiveness	0.20	0.52	0.02	
Maternal demandingness	−0.12	0.32	−0.01		Maternal demandingness	−0.14	0.36	−0.02	
					Bonding	−0.25	0.24	−0.05	
					Resilience	0.03	0.23	0.01	
					Social competence	0.42	0.22	0.08^*^	
					Recognition of positive behavior	0.11	0.22	0.02	
					Emotional competence	−0.48	0.23	−0.09^*^	
					Cognitive competence	0.16	0.25	0.03	
					Behavioral competence	−0.68	0.25	−0.12^**^	
					Moral competence	−0.13	0.23	−0.02	
					Self-determination	−0.31	0.24	−0.06	
					Self-efficacy	0.16	0.20	0.03	
					Clear and positive identity	0.58	0.23	0.13^*^	
					Beliefs in the future	−0.42	0.20	−0.10^*^	
					Prosocial involvement	−0.01	0.23	−0.00	
					Prosocial norms	−0.12	0.21	−0.02	
					Spirituality	−0.26	0.12	−0.08^*^	

* *p < 0.05*,

** p < 0.01, and

*** *p < 0.001*.

Model 2 of Table 3 shows the results of the multiple regression analysis when PYD attributes and parenting behavior were simultaneously input into the model as predictors. It was revealed that after introducing the 15 PYD attributes, none of the parenting behaviors predicted adolescent SNA. On the other hand, four PYD attributes—emotional competence (β = −0.09; *p* < 0.01), behavioral competence (β = −0.12; *p* < 0.001), belief in the future (β = −0.10; *p* < 0.01), and spirituality (β = −0.08; *p* < 0.01)—were negatively associated with SNA, indicating that they may mitigate adolescents' SNA. Two PYD attributes, social competence (β = 0.07; *p* < 0.05) and clear and positive identity (β = 0.13; *p* < 0.001), were positively related to SNA. Participants with higher scores on social competence and clear and positive identity reported more problematic social networking behavior. The PYD attributes explained 7% of the SNA variance, indicating a small effect size.

**Figure 1:**
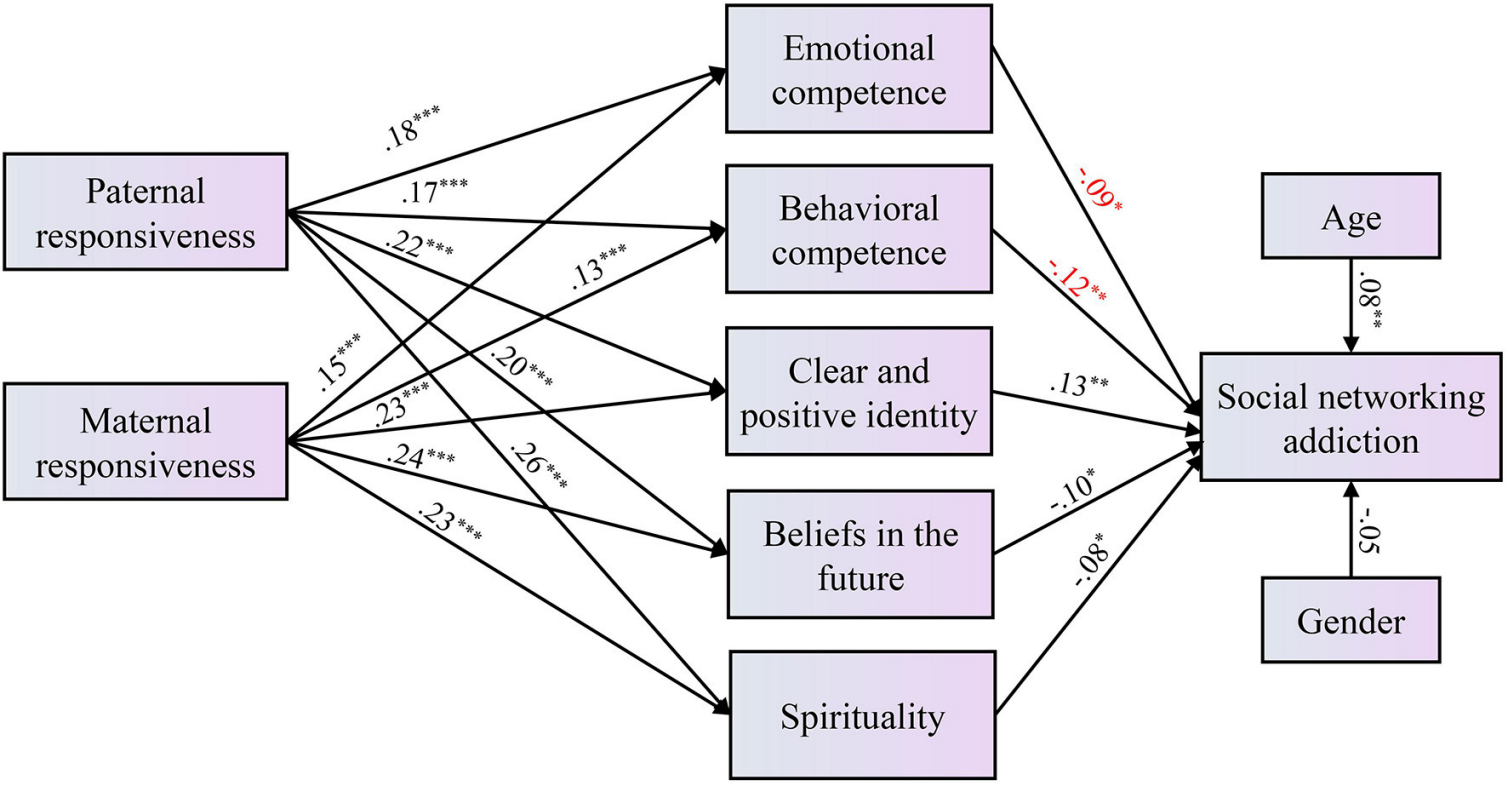
Final model of the relationships among parenting behavior, PYD attributes and social networking addiction.

Results of the regression analyses examining the effects of parenting behavior on the 15 PYD attributes are summarized in Table 4. Both paternal responsiveness and maternal responsiveness were positively related to all 15 PYD attributes, indicating that parental responsiveness may be a precursor of adolescents' positive development in multiple areas. Paternal demandingness was found to be negatively associated with adolescents' cognitive competence, and maternal demandingness was positively related to adolescent prosocial norms. These findings suggest that both paternal and maternal responsiveness have indirect effects on adolescent SNA through the full mediation of PYD attributes. The whole model explained 8% of the variance in SNA. We further illustrate this mediation model in Figure 1.

**Table 4:** Regression of parenting behavior on PYD attributes.

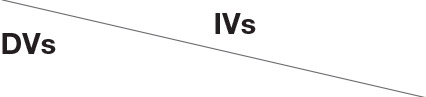	**Paternal responsiveness**			**Paternal demandingness**			**Maternal responsiveness**			**Maternal demandingness**		

	**B**	**SE**	**β**	**B**	**SE**	**β**	**B**	**SE**	**β**	**B**	**SE**	**β**
Bonding	0.62	0.07	0.28^***^	0.00	0.05	0.00	0.54	0.07	0.23^***^	0.02	0.05	0.01
Resilience	0.32	0.08	0.14^***^	−0.00	0.06	0.00	0.56	0.08	0.23^***^	−0.05	0.06	−0.03
Social competence	0.40	0.08	0.18^***^	−0.02	0.05	−0.01	0.31	0.08	0.13^***^	0.01	0.06	0.01
Recognition of positive behavior	0.54	0.08	0.23^***^	0.02	0.06	0.01	0.37	0.09	0.15^***^	−0.01	0.06	−0.01
Emotional competence	0.43	0.08	0.18^***^	−0.05	0.06	−0.03	0.38	0.09	0.15^***^	0.04	0.06	0.03
Cognitive competence	0.45	0.08	0.21^***^	−0.11	0.05	−0.08^*^	0.37	0.08	0.16^***^	0.00	0.06	0.00
Behavioral competence	0.35	0.07	0.17^***^	−0.02	0.05	−0.01	0.29	0.08	0.13^***^	0.02	0.05	0.01
Moral competence	0.29	0.08	0.14^***^	−0.01	0.05	−0.00	0.41	0.08	0.17^***^	0.06	0.06	0.04
Self-determination	0.46	0.08	0.20^***^	−0.00	0.06	−0.00	0.37	0.08	0.15^***^	−0.03	0.06	−0.02
Self-efficacy	0.42	0.08	0.18^***^	−0.00	0.06	−0.00	0.43	0.09	0.17^***^	−0.02	0.06	−0.01
Clear and positive identity	0.59	0.09	0.22^***^	−0.01	0.06	−0.01	0.66	0.10	0.23^***^	−0.11	0.07	−0.06
Beliefs in the future	0.53	0.09	0.20^***^	0.04	0.06	0.03	0.70	0.09	0.24^***^	−0.08	0.07	−0.04
Prosocial involvement	0.50	0.08	0.21^***^	0.04	0.06	0.02	0.51	0.08	0.20^***^	0.01	0.06	0.01
Prosocial norms	0.33	0.08	0.15^***^	−0.00	0.05	−0.00	0.36	0.08	0.15^***^	0.16	0.06	0.10^**^
Spirituality	0.91	0.12	0.26^***^	0.02	0.08	0.01	0.87	0.13	0.23^***^	−0.04	0.09	−0.01

* *p < 0.05*,

** p < 0.01, and

*** * p < 0.001*.

## Discussion

This study is one of the first to investigate adolescent SNA and its associated factors from a positive youth development and family perspectives. The findings show that excessive use of social networking sites and applications is quite common among Hong Kong adolescents. After controlling for age and gender, four PYD attributes were negatively associated and two PYD attributes positively associated with the participants' SNA. Regarding parenting behavior, higher levels of paternal and maternal responsiveness were related to higher levels of PYD attributes, which further associated with lower levels of SNA among adolescents.

Based on the BSMAS, 11.4% of the participating adolescents could be classified as having SNA. Using a self-reported measure, nearly 20% perceived themselves as “fairly” or “strongly” addicted to SNSs. This result was largely consistent with previous studies indicating that 10–20% of adolescents are at risk of SNA [e.g., (6)]. While SNA seems less prevalent in adolescent groups than in university students, our findings suggest that many Hong Kong teenagers have difficulty controlling their SNS use despite its negative impact on their lives and studies; furthermore, some have developed the habit of using SNSs as a coping strategy to escape from problems. In the study, 29.7% of the students reported spending 2–3 h on SNSs daily, and 18.6% reported spending over 4 h a day on SNSs, despite their limited freedom and time for using electronic devices compared to university students and young adults. Longitudinal studies have demonstrated strong links between longer screen time (above an hour a day) and lower psychological well-being in young people including less curiosity, attention, self-control, emotional stability, and a higher probability of an anxiety and depression diagnosis (54). Consequently, Hong Kong adolescents' SNS overuse and associated long screen time deserve more serious public and research attention, with a need for the development and implementation of effective strategies and clear guidelines on adolescents' healthy SNS use.

Consistent with our hypothesis that PYD attributes mitigate adolescents' problematic SNS use; emotional competence, behavioral competence, beliefs in the future, and spirituality were found to be negatively associated with SNA. The literature supports the role of emotional competence in both general internet addiction and SNA. Highly emotionally competent individuals understand and manage their emotions effectively and thus are less likely to use SNSs to cope with negative emotions and to distract themselves. Researchers also report that young people suffering from depression and anxiety have a high risk of developing SNA (55), indicating the protective effect of emotional regulation on SNA development. Behavioral competence, defined as the ability to make effective behavioral choices and perform socially acceptable behavior, is related to self-control, an important predictor of problematic behavior (36, 56). In behavioral addiction literature, poor self-control and deficient self-regulation are considered a major cause of addictive internet and social media use (26, 57). Individuals with little self-control are primarily governed by immediate gratification and short-term goals and are thus more prone to SNA. Enhancing behavioral competence would enable adolescents to control impulsivity, make behavioral choices wisely, delay gratification, and pursue long-term rewards, so contributing to SNA prevention.

We also found belief in the future and spirituality to be negatively associated with SNA. These two PYD attributes are pertinent to a belief in a higher power, a sense of life meaning, values regarding life choices, having valued and attainable goals, and positive expectancies for the future. On one hand, highly spiritual adolescents and those with positive beliefs in the future are more likely to have a purpose in their life and long-term goals, motivating them to discipline themselves, to focus less on immediate rewards, and to control urges to constantly use SNSs. On the other hand, as with religiosity (58, 59), spirituality may help deter problematic behavior such as SNA through its normative (i.e., problematic behavior is against religious norms) and integrative (i.e., social support from other members of the same religious group) functions, that may result less in resorting to SNSs to reduce negative emotions. Previous studies have indicated that spirituality and hope are negatively related to depression and anxiety, two precursors of addictive behavior (60, 61). Our findings suggest that promoting spiritual development and helping adolescents establish beliefs in the future may help reduce and prevent SNA.

Unexpectedly, social competence and a clear and positive identity were positively related to adolescents' SNA. Previous studies on these attributes' effects on internet addiction have generated mixed findings. For example, Caplan (20) illustrated that people with low levels of social competence tend to perceive online communication as less threatening than face-to-face social interaction, thus developing a preference for computer-mediated communication, leading to excessive or compulsive internet use. Yet, in a large-scale study (62), heavier SNS use was positively linked to offline social competence among 16 and 17 year-olds. Online communication could provide a space for adolescents to obtain social support and explore peer relationships. Interaction via SNSs also allows adolescents to practice their social skills before applying them face-to-face (63). Similarly, in a more recent study examining the relationship between cyberbullying and emotional engagement in school (64), researchers proposed that students who spend more time on social media may have larger social networks and more friends at school; they may also be more popular and accepted while at the same time be more at risk to cyberbullying. It would be interesting to look further into the mutual effects between social networking behavior and social competence in future studies.

A clear and positive identity is conceptualized in the PYD framework as self-esteem, exploration, and commitment to self-definition (32, 65). Several studies have found a negative relationship between self-esteem and internet addiction. Joinson (66) reported that people with low self-esteem preferred e-mail communication to traditional interpersonal communication. Another study revealed greater instant messaging use among young people with low self-esteem (67). Our finding that a clear and positive identity correlates positively with SNA seems to contradict these reports. However, the current study is based on cross-sectional data. SNS usage could help adolescents to develop a clear and positive identity through establishing supportive peer relationships, particularly important for adolescents' healthy identity formation and social development. The causal effects of a clear and positive identity on SNA demand further longitudinal investigation.

We examined the role of positive parenting behavior in adolescents' SNA and differentiated the respective effects of paternal and maternal behavior. It was revealed that while parenting behavior did not exert direct effects on adolescent social networking addiction, both paternal and maternal responsiveness were negatively associated with SNA through the full mediation of PYD attributes. In other words, when parents are more involved and responsive, their children are more likely to develop various positive attributes such as emotional competence, behavioral competence, and a positive belief in the future; attributes that reduce young people's problematic social networking behavior. This finding is compatible with the relational developmental system theory (34) illustrating that PYD attributes are developed in supportive relationships and contexts, and that the family is the most important environment to provide such relationships in supporting youth healthy development.

It is worth noting that although previous studies generally demonstrate that maternal emotional support and good mother-child relationships lead to positive outcomes in children (68, 69), we demonstrated significant relationships between both parents' responsiveness and the adolescents' SNA. In fact, recent studies on parenting in Hong Kong suggest that the traditional roles of Chinese fathers and mothers have changed in contemporary Hong Kong society, with mothers perceived to be more demanding and strict than fathers, although fathers were still perceived as more distant and less involved in child education than mothers (70). In a recent survey based on 1,510 Hong Kong families, researchers reported that fathers and mothers did not differ in parental warmth, but that the mother-child relationship was stronger than the father-child relationship (71). Our finding that paternal responsiveness significantly associated with the development of different PYD attributes further supports the important role of fathers. Given the relatively low paternal involvement in parenting, it is suggested that more strategies are needed to support and empower fathers to actively participate in their child's education to promote the healthy development of young people.

Neither maternal nor paternal demandingness affected the adolescents' SNA, even though prior research has shown that parental monitoring and supervision of children's behavior is associated with adolescents' decreased involvement in norm-breaking behavior (72). Perhaps parents' specific mediation behavior (i.e., the methods parents adopt to mitigate children's SNS use) have more influence on adolescents' SNA than do general parenting styles. For example, Chang et al. (73) found that adolescents reporting higher levels of parental restrictive mediation (i.e., using explicit rules about children's internet use) were less likely to develop internet addiction or to engage in cyberbullying. Future studies on parenting and adolescent SNA should investigate both general parenting styles and specific parenting practices.

This study has several limitations. First, while we did not intend to propose a full explanatory model of the development of social networking addiction, the variance in SNA explained by our current predictors was not high, indicating that there are other important factors contributing to the formation of SNA that have not been included in the present study. For example, specific biological and psychosocial elements that determine an individual's vulnerability to SNA (e.g., high compulsivity, emotional distress), maladaptive cognition about SNS use, and expectations of positive outcomes associated with SNS use (74, 75). It would be useful to examine how different protective factors may interfere with or mitigate the influence of these risk factors in preventing the development of SNA.

Second, our cross-sectional design precludes detailed insight into causal relationships among the investigated variables. Longitudinal studies should be conducted to examine whether positive youth development attributes and parenting behavior can reduce social networking addiction in adolescents. Third, as discussed earlier, we only focused on the effects of general parenting behavior on adolescents' SNA; the roles of specific parenting practices such as SNS-related mediation and parental SNS use should be further investigated. Fourth, the study was based on a sample of Hong Kong adolescents, and the findings' generalizability to other cultures requires scrutiny, especially given the varying roles of fathers and mothers in children's socialization across cultures. Finally, both SNS usage and the predictors were measured solely on the adolescents' self-reported data, and a social desirability bias might not have been completely prevented. Future studies could collect information from parents.

It should be pointed out that the data reported in the present paper were collected between February and June in 2019 before the COVID-19 outbreak. During the pandemic, social media has become the major channel for people to obtain updated information related to COVID-19 and to keep connection with their important others. Consequently, there has been a surge in social media usage during the nation-/city-wide lockdown as reflected in recent literature (76). This new phenomenon has attracted growing research attention; scholars and practitioners start to rethink about the definition of SNA: “whether this compulsive use of social media is just a ‘phase' and a coping mechanism or an indication of addictive behavior having mental health implications” ((77), p. 1). There is a great need to take into account the particular contextual issue of global pandemic when examining people's use of SNSs and the associated factors.

Regarding the present finding, it would be worthwhile to investigate the moderating effects of different PYD attributes (e.g., self-esteem, spirituality, bonding) as well as positive parenting behaviors on the relationship between adolescents' excessive use of SNSs and their psychosocial well-being during the pandemic. In fact, a recent study revealed that individuals with approach coping style reported more social networking activities and perceived more family support, which was associated with lower subsequent levels of depression (68). This suggests the protective role that positive attributes and positive relationship may play in enabling individuals to use SNSs in an adaptive way. Future research could further study the factors that differentiate adaptive from maladaptive use of SNSs among adolescents, which may provide new intervention directions. Besides, as there are mixed findings on the influence of PYD on adolescent problem behavior (78, 79), further research that probes into the specific effects of various PYD attributes on youth problems and the associated mechanisms should be conducted.

## Conclusion

Despite the limitations, the present findings have theoretical and practical implications. Theoretically, based on a PYD approach, this study identified protective factors at the individual (PYD attributes) and family levels (parental responsiveness) that may shield adolescents from developing SNA. This increases our understanding of adolescent SNA and could contribute to the development of a more comprehensive theoretical model of SNA formation comprising both protective and risk factors.

Practically, while it is unlikely that young people will stop using SNSs in a fast paced electronic era, helping them to better manage their SNSs use and preventing the development of SNA is more important, timely, and cost-effective than focusing on the treatment of a small group of young people with SNA. Our findings support the significant role not only of mothers, but also of fathers in adolescent positive development, providing evidence for policy-makers and practitioners in Hong Kong to further encourage and facilitate fathers' involvement in child education. For example, family services should also aim to support fathers in effective parenting strategies. As fathers are still often the primary breadwinner in the family, the government may consider developing strategies to support working fathers to be more involved in their child's life.

More importantly, the identified PYD attributes conducive to SNA reduction in the present study (i.e., emotional competence, behavioral competence, belief in the future, spirituality) suggest new directions for SNA prevention programs. First, helping professionals should be aware of the protective effects of different positive attributes on adolescents' SNA, assess the development of these attributes among their clients, and help them to uncover and leverage these strengths when using SNSs. Second, SNA prevention programs should incorporate training components targeting at promoting emotional competence and behavioral competence for adolescents. Third, practitioners should teach parents and educators not only the negative effects of overuse of SNSs, but the potential benefits of extensive social networking activities, especially during the current pandemic. Updated research findings and skills on how to use SNSs adaptively should be further disseminated. Research shows that although life skills and social competencies are regarded to be important for adolescent development, its coverage is not adequate in the formal curriculum (80).

We hope these preliminary findings will motivate researchers to adopt a more positive framing perspective for investigating SNA and other addictive behavior in young people.

## Data Availability Statement

The raw data supporting the conclusions of this article will be made available by the authors, without undue reservation.

## Ethics Statement

The studies involving human participants were reviewed and approved by the Human Subjects Ethics Committee of the Hong Kong Polytechnic University. Written informed consent to participate in this study was provided by the participants' legal guardian/next of kin.

## Author Contributions

LY conceptualized and designed the study, collected the data, interpreted the data, drafted the manuscript, and approved the final manuscript as submitted. DS designed the study, interpreted the data, drafted the manuscript, and approved the final manuscript as submitted. Both authors contributed to the article and approved the submitted version.

## Conflict of Interest

The authors declare that the research was conducted in the absence of any commercial or financial relationships that could be construed as a potential conflict of interest.
